# Establishing Smoke-Free Homes in the Indigenous Populations of Australia, New Zealand, Canada and the United States: A Systematic Literature Review

**DOI:** 10.3390/ijerph14111382

**Published:** 2017-11-14

**Authors:** Leah Stevenson, Sandy Campbell, India Bohanna, Gillian S. Gould, Jan Robertson, Alan R. Clough

**Affiliations:** 1 College of Public Health, Medicine and Veterinary Science, James Cook University, Cairns 4870, Australia; sandy.campbell@jcu.edu.au (S.C.); india.bohanna@jcu.edu.au (I.B.); jan.robertson@jcu.edu.au (J.R.); alan.clough@jcu.edu.au (A.R.C.); 2 Australian Institute of Tropical Health and Medicine, James Cook University, Cairns 4870, Australia; 3 School of Medicine and Public Health, The University of Newcastle, Newcastle 2300, Australia; gillian.gould@newcastle.edu.au

**Keywords:** Indigenous populations, smoke-free homes, second-hand smoke, tobacco prevention, Oceania, America

## Abstract

A smoke-free home can have multiple benefits by reducing exposure to secondhand smoke (SHS), supporting quit attempts among active smokers, and discouraging adolescents from taking up smoking. The aim of this review was to summarize the literature on the establishment of smoke-free homes in Indigenous populations and identify the supporting influences and barriers, using the Social Cognitive Theory lens. A search of the Medline, CINAHL, Cochrane Collaboration and PyscINFO databases and manual searches of relevant peer-reviewed literature was completed, focusing on Indigenous populations in developed economies of North America and Oceania. Of 2567 articles identified, 15 studies were included. Ten studies included Indigenous participants only, and of these just three focused entirely on SHS in the home. Knowledge of the harms associated with SHS was the most common theme represented in all the studies. This knowledge fueled parents’ motivation to protect their children from SHS by establishing smoke-free homes. Individuals who approached implementation with confidence, coupled with clear communication about smoke-free home rules were more successful. Barriers included challenges for families with multiple smokers living in the same dwelling. There is limited research regarding managing smoking behaviors in the home among Indigenous populations, even though this approach is a successful catalyst for smoking prevention and cessation. Research to understand the influences that support the establishment of smoke-free homes is required for better-informed intervention studies.

## 1. Introduction

Worldwide, secondhand smoke (SHS) from tobacco is one of the most common indoor pollutants and many children are exposed in the family home [1]. In Australia, 20% of Indigenous children have at least one parent that smokes inside the home compared to 7% of children in non-Indigenous households [2], In New Zealand, a 2012–2013 national survey showed Maori children were 2.6 times more likely to be exposed to SHS in both homes and cars compared to children from the general population [3]. Surveillance in Canada and the United States indicates that both Canadian First Nations peoples and Native American children are two to three times more likely to be exposed to SHS in the home than the general population [4,5]. Indigenous children in all four countries experience higher SHS exposure than non-Indigenous children [2,3,4,5]. Children exposed to secondhand smoke have an increased risk of asthma, lower respiratory tract infections and are at an increased risk of sudden infant death syndrome [1].

Smoking rates are higher among the First Peoples compared to the rest of the population in colonised countries around the world; and higher among First Peoples residing in rural and remote communities [6]. New Zealand Maori people have reduced daily smoking rates consistently over the past decade to 35.5% however is still higher than the general population at 14.2% [7]. In Australia, Aboriginal and Torres Strait Islander people living in remote areas are more likely to be daily smokers (50%) compared to those living in non-remote areas (38%) [8]. Recently, smoking rates among Indigenous Australians living in urban centers have shown promising decreases, however, little has changed in remote areas [8]. Similarly, Canadian First Nations people living “on-reserve” (areas set aside for First Nations people, generally in rural areas) have higher smoking rates than those living “off-reserve” [9]. Smoking rates among American Indian and Alaskan Natives are 29.2%, and are also higher than the general population at 16.8% [10]. This review has focused on these populations as they share similar historical experiences, particularly from the 19th century when Indigenous peoples were either forced into the lowest social classes, or removed from their lands into reserves or missions [11]. This has contributed to ongoing negative impacts on Indigenous tradition, society, economy and health [11].

Smoke-free homes have been recognized as an effective strategy for protecting non-smoking adults and children against SHS [12]. While advice to parents about smoking cessation alone does not increase the protection of children from SHS, supporting a smoker to implement a smoke-free home can potentially improve the air quality in the home environment, and increase parental quit attempts [12,13]. Research has documented that a strong history of past quit attempts predicts successful quitting [14]. A systematic review of the exposure of young people to smoking behaviors of others, and their own subsequent smoking behavior concluded that home restrictions remain important for reducing the uptake of smoking among adolescents [15]. Supporting the establishment of smoke-free homes, shown to be effective for prevention of uptake and cessation of tobacco smoking in the general population, may be a particularly useful intervention for Indigenous populations [15]. 

This review utilizes Social Cognitive Theory (SCT) to inform health promotion practices for establishing a smoke-free home. SCT is used widely in health promotion and addresses the interaction between an individual’s characteristics and their behavior with an emphasis on individual self-efficacy and collective efficacy [16,17]. Self-efficacy is described as one’s individual ability to succeed in achieving a certain behavior, while collective efficacy refers to the ability of a group of people to control the behavior of individuals and groups [16,17]. Personal characteristics and behaviors are influenced by, and can in turn influence both social and physical environments [16]. These reciprocal factors, central to the theory, are described as personal, behavioral and environmental (physical and social environments) influences [16]. SCT has been recommended by researchers to develop an understanding of the relationship between characteristics of household members and smoking restrictions in their homes [18,19], however, it has been used in very few studies. The exceptions include a protocol for a smoke-free home intervention with Australian Aboriginal and Maori people by Johnston et al. [20] and a study with people living in low socio-economic conditions in a rural setting in the United States [21]. With exposure to SHS in the home being higher in Indigenous populations, it is timely to consider the range of social and physical environmental factors that influence a person’s smoke-free home behaviors. This review aims therefore to understand personal, behavioral and environmental influences on establishing smoke-free homes to assist in the development of smoke-free home interventions for Indigenous families in four high-income countries. 

## 2. Methods

### 2.1. Search Terms

**Figure 1:**
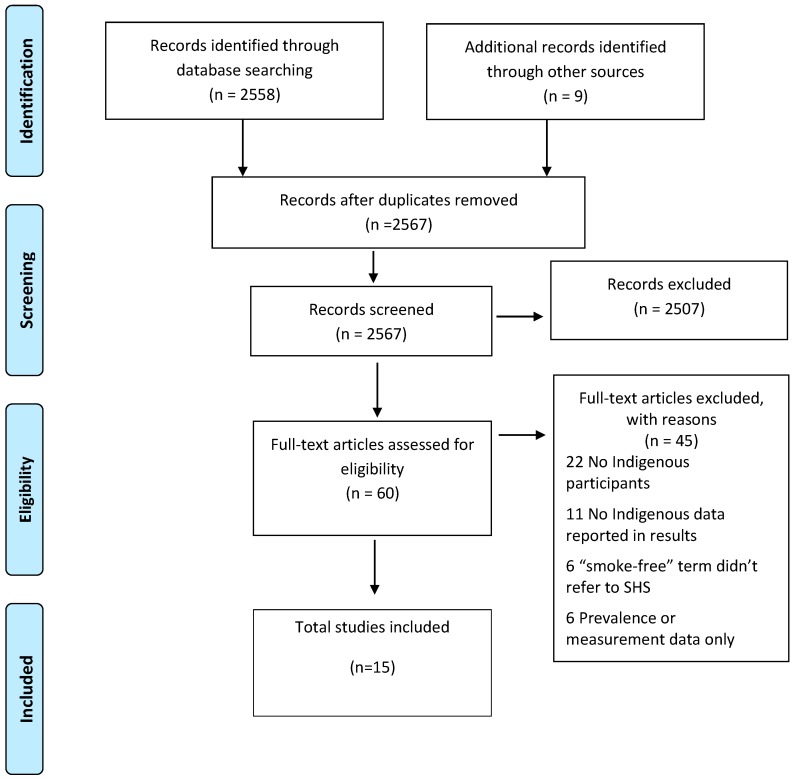
Summary of the article selection process as recommended by the PRISMA statement.

In February 2017, databases of Medline via Ovid, CINAHL, Cochrane Collaboration, PsycINFO were searched. The databases were searched in an iterative process to capture different sets of journals and maximize completeness of the review. Searches used a combination of the following keywords: (“smoke-free” OR “passive smoking” OR “environmental tobacco smoke” OR “second hand smoke”) combined with (“Indigenous” OR “Native” OR “Ethnic” OR “Aboriginal” OR “Torres Strait Islander” OR “Maori” OR “First Nation” OR “Native American” OR “Alaska Native” OR “Inuit” OR “Metis” OR “Pacific Islander” OR “Native Hawaiian”). Only peer-reviewed studies were included, but of all methodology types, with no restrictions on year of publication. Reference lists of eligible articles were searched for additional publications. We scanned 2567 titles. Duplicates, and studies that did not satisfy the inclusion criteria, were removed. Manuscript titles were screened by LS. If the title did not provide sufficient information to identify its relevance to meet the selection criteria, the abstract was reviewed. Where the abstracts did not clearly inform the exclusion criteria, the full paper was read. To ensure the process was thorough, sixty complete articles were independently checked (by AC and LS) (Figure 1). 

### 2.2. Inclusion Criteria

The populations identified for this review include Indigenous peoples from high-income countries colonized by Europeans: Australia, New Zealand, Canada and the United States. Studies were included if they reported on Indigenous peoples’ attitudes, behaviors and experiences of managing SHS within a home with one or more families residing. 

### 2.3. Exclusion Criteria

Studies were excluded from the review if: there were no Indigenous participants or a proportionately very low number participated in the study (for example studies where Indigenous people were not the main focus of the study); or if the paper included Indigenous participants but the results were not reported separately from the general population; the term “smoke-free” was used to refer only to an individual quitting or being a non-smoker, rather than SHS in the home; or if the study only reported prevalence data. 

### 2.4. Data Extraction and Synthesis

**Figure 2:**
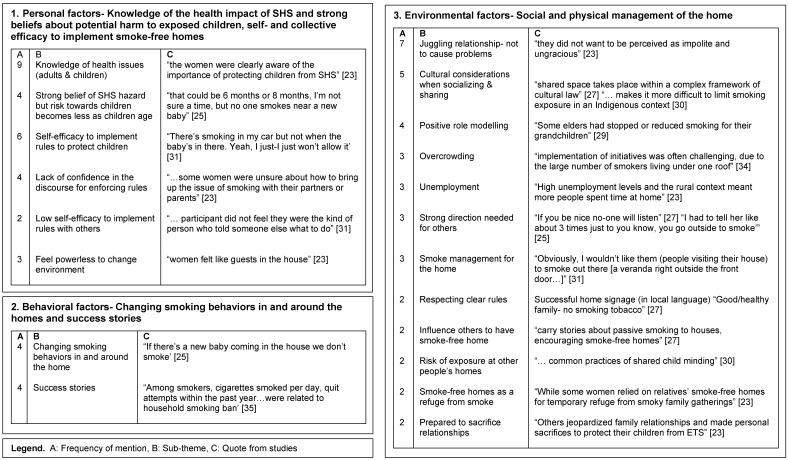
Indigenous specific themes arranged into the three reciprocal determinism factors of the Social Cognitive Theory from the 15 included studies.

Data was extracted for each included study (LS) and organized and managed using an Excel spreadsheet. Columns included study design, study aim, country and locality, Indigenous-specific or other population group, and summary of study results (Table 1). A deductive approach was used to map the data from the results sections of the papers into SCT factors of personal, behavioral and environmental influences by LS. Following this, an inductive thematic analysis was undertaken by three researchers (LS, AC and IB), who independently coded the data from the three reciprocal influences, and suggested emergent themes. Agreement about interpretation of the extracted data and themes was achieved as a group by discussion and consensus. Key study results are arranged according to SCT factors, with representative quotes to illustrate the themes (see Figure 2). Only data about the Indigenous populations was extracted from the articles. 

## 3. Results

Fifteen papers met the inclusion criteria and were incorporated into this review (Figure 1). Ten studies were conducted exclusively in Indigenous populations, and the remainder involved mixed populations (i.e., Indigenous and non-Indigenous). Seven studies were conducted in Australia, three in New Zealand, two in both Australia and New Zealand, two in Canada and one in the United States. Eight studies were qualitative and six were quantitative, and one study used multi-methods. A range of data collection methods were used. Six studies used interviews, two used focus groups, four used interviews and focus groups and three studies used quantitative surveys. Six studies focused on female participants while they were pregnant or post-partum, two studies recruited parents and caregivers, and one study investigated the attitudes of children. Samples in the remaining six studies consisted of general community members, health staff and/or key informants. The total number of participants in the studies was 5238, and of these, 3140 (59.9%) were Indigenous participants.

The themes identified from this review were organized into the three reciprocal determinism factors of SCT; personal, behavioral and environmental factors. These include: Personal factors—Knowledge of the health impact of SHS, strong beliefs about potential harm to exposed children and self-efficacy and collective efficacy to implement smoke-free homes; Behavioral factors—Moving smokers outside or removing children from the smoky environments were the main behaviors to reduce SHS exposure; Environmental factors—Social and physical management of SHS in the home. Figure 2 shows Indigenous specific themes from the 15 included studies arranged into the three reciprocal factors of SCT. 

### 3.1. Personal Factors—Knowledge of the Health Impact of SHS, Strong Beliefs about Potential Harm to Exposed Children and Self-Efficacy and Collective Efficacy to Manage Smoke-Free Homes

Knowledge of the health risks associated with SHS was the most represented theme among participants across the studies. Responses by both adults and children indicated high levels of knowledge about the risks of SHS for children [22,23,24,25,26,27,28,29,30]. Four studies reported that participants had concerns for young children exposed to SHS, and a good understanding as to why it was important to protect them [23,25,26,31].

Strong beliefs about the harm to children from SHS exposure was a prominent personal influence, and the need to protect newborn babies was highlighted [25,31]. However, there was an age threshold where community members believed it became more acceptable to smoke near young children. These ages were reported in one study as being close to two months old, and six to eight months old in another [25,30]. A study by Kegler and Malcoe showed that caregivers with strong beliefs towards the harms of SHS were four times more likely to report total smoking restrictions in their homes, compared to those who possessed weaker beliefs [35]. These studies demonstrate that strong beliefs about protecting children from SHS can be important motivators for the establishment of smoke-free homes. 

#### Self- and Collective Efficacy

High self-efficacy or self-belief in the ability to succeed, is considered a cornerstone to behavior change [18]. Both high and low self-efficacy were discussed in several studies [23,25,26,28,30]. Pregnant women were reported as being highly motivated and confident in ensuring they reduced tobacco intake and SHS exposure [25,30]. Being pregnant also appeared to be a time of strength to promote smoking bans in the home [25,30]. In a study by Bottorff et al., female participants suggested that those who could implement home smoking rules were strong and confident characters, and these characteristics were essential to successfully administer non-smoking rules [23]. “My house, my rules” [25] was a strong directive by an Indigenous Australian mother when describing her approach to implementing smoke-free home rules. Collective efficacy was discussed by children, parents and community members [23,27,28]. Children expressed that they had confidence to act to reduce their own risks or to protect their siblings from SHS [23]. In a large remote community-based study, some community members expressed a desire to implement smoking bans in all homes, community areas and streets [27]. 

### 3.2. Behavioral Factors—Changing Smoking Behaviors in and around the Home and Success Stories

#### 3.2.1. Changing Smoking Behaviors in and around the Home

The smoking and non-smoking behaviors in and around the home were reported in several studies and participants described a range of approaches [25,31,35,36]. Glover et al. described how young mothers managed SHS collaboratively with their partners or other family members by removing smokers from the home [31]. A study by Walker et al. found that although there was high self-reporting of smoke-free home environments, children were still exposed to second-hand smoke through underreporting of exposure or inadequate smoke-free home rules [36]. Parents described how they may refuse to enter homes of friends or families with their children, until the smokers left the house [31]. In one study, participants described how guests at a home might choose to vacate while others were smoking inside [25]. It was also reported in two quantitative studies that home smoking restrictions were associated with smoking less and an increased number of quit attempts by smokers [28,35].

#### 3.2.2. Success Stories

There were success stories among Indigenous people living in challenging socio-economic circumstances when implementing smoke-free home environments [22,23,27]. Indigenous people living in low socio-economic conditions where smoking rates were high in rural Canada, United States and Australia, gave encouraging accounts of personal experiences [23,27,35]. Reducing SHS exposure in the home was achieved by managing the smoking behaviors of visitors. Importantly, one study found that half of low-income homes occupied by Native Americans had total smoking bans [35]. Findings from this quantitative study also showed significant associations between the number of cigarettes smoked per day, quit attempts and strong belief towards the harms of SHS to children and babies [35].

### 3.3. Environmental Factors—Social and Physical Management of the Home

#### 3.3.1. Relationships

Seven studies reported that it was problematic for people to implement smoke-free home rules if protecting relationships took priority. Participants believed it was important to avoid appearing rude to those who smoked [23,24,25,27,31,34]. Current smokers were more likely to agree that it could be perceived as rude to ask guests not to smoke inside the home compared to non-smokers [32]. In three studies, participants expressed that it was of great concern to not jeopardize relationships by being too challenging in one’s expression [23,25,27]. In contrast, two studies reported that a strong belief in the harm of SHS was enough for some individuals to risk challenging their relationships, in order to protect children from harm [23,26].

Cultural considerations in relationships when implementing smoke-free homes were discussed in five studies [23,27,29,30,34]. The tradition of sharing tobacco while socializing was an important theme. A study by Robertson et al. described the important cultural practice where shared community space was managed through kinship systems. This means that cultural protocols need to be considered when communicating with others about managing land or spaces, including the family home [27]. Three studies reported the importance of socializing with others in the community, and that smoking was often ingrained into this practice [23,30,34].

#### 3.3.2. Communicating Smoke-Free Rules

Six studies described residents’ attempts to communicate smoke-free home rules to their guests [23,24,25,27,31,33]. Participants in two studies discussed how “no smoking” signage was created for homes to communicate their rules [27,31]. In another study, focus group participants explained how diplomatic conversations were necessary, but not always successful [23]. Two studies highlighted a lack of knowledge about how to broach the subject with others in the household, which made it difficult to implement smoke-free home rules [23,25]. Three studies provided evidence that enforcing strict rules, and giving strong instructions, was necessary to prevent visitors or family members from smoking in the home. This method of creating clear, direct rules was deemed more successful in ensuring that others adhered to the rules [23,25,27]. 

#### 3.3.3. Role Modelling

Three studies reported that it was important to people that they be good role models for adolescents and young children [22,29,31]. Parents used smoking restrictions in the home as a method for discouraging children from taking up smoking, in addition to not smoking in front of them, and avoiding smoky public spaces [22]. One study identified that where smoking had not been allowed in their own childhood homes, parents often decided to keep the rule for their own children [31]. Role modelling by Elders and community members was valued, and it was hoped that Elders would actively show leadership around smoking issues [29].

#### 3.3.4. Physical Management of SHS in the Home

The way people managed the physical space to reduce SHS within the home was reported in several studies [23,25,28,31]. A study by Gould et al. reported that some mothers with new born babies would allow smoking in a specific room in their house, away from the children [25]. Participants in two studies described verandahs or other external spaces as appropriate smoking areas in their smoke-free home management [28,31]. Some smoke-free homes became a refuge for people who wanted to avoid smoky environments elsewhere [23,25]. 

#### 3.3.5. Socio-Economic Issues

Unemployment, overcrowding and stress were key issues facing some Indigenous families who found it challenging to create smoke-free homes [23,34]. Implementing smoke-free homes was difficult because there were often many smokers living in households [34]. High levels of unemployment often led to more people spending extended periods of time in the house smoking, and overcrowding of living spaces exacerbated the issue [23]. 

## 4. Discussion

This review of SHS in the home and implementing smoke-free homes among Indigenous peoples in four high-income countries of Australia, New Zealand, Canada and the United States of America comprised fifteen included studies. The review analyzed the range of factors that influence the implementation of smoke-free homes. The results of the review were organized into the three reciprocal determinism factors of SCT; personal, behavioral and environmental factors [17]. 

The key personal factors influencing smoke-free home behaviors were related to individuals’ knowledge of the health impacts of SHS, strength of beliefs about potential harm to exposed children and also levels of self-efficacy or self-belief in the ability to succeed at implementing a smoke-free home. Collective efficacy was demonstrated in some studies by the shared belief to reduce secondhand tobacco smoke exposure in the home. Important behavioral factors included children removing themselves from the exposure in the home or parents establishing rules where smoking was not to occur in the home. Environmental factors were associated with both physical and social environments. They included how individuals and groups went about the physical management of SHS in the home, approaches to communicating smoke-free rules, how relationships were managed, role modelling and socio-economic issues. No papers in this review considered the reciprocal nature of personal, behavioral and environmental factors that influenced the establishment of smoke-free home environments. However, this review has identified potential links between each of the SCT factors that could promote a smoke-free home environment. Programs aimed at promoting smoke-free home environments in Indigenous communities may be strengthened by consideration of each of the reciprocal determinism factors during design and development of interventions, and how these may interlink. 

It is well recognized by health promotion practitioners, that knowledge about health and health risks is vital, however, it does not ensure positive behavior change [37]. Studies have shown that high awareness of the significant negative effects of SHS exposure is not always linked to behaviors that result in smoke-free home environments [38]. Our review has demonstrated this is not dissimilar among Indigenous populations. From our review, there appeared to be social norms operating among some groups that allow smoking in the home, potentially hampering collective efficacy efforts by families to establish smoke-free homes. For example, some parents felt strongly about not smoking near newborn babies, but smoking around slightly older infants was of less concern [25,30]. Factors such as crawling and toddling infants being too mobile to leave unattended inside the home has been reported to make it difficult for some parents to go outside to smoke [39]. At the same time however, there was evidence that such social norms are being challenged within some Indigenous communities [25]. Importantly, a study of Aboriginal women in small reserve communities in Canada, found a growing social norm towards smoke-free homes [23]. 

In addition to knowledge of health risks, high personal self-efficacy has been shown to enhance motivation, goal setting and commitment to achieving positive outcomes [40]. This review found many families had skills to manage physical space in the home, however, a range of social issues affecting households can hinder efforts to establish smoke-free homes [23]. Socio-economic contexts are important considerations in smoke-free home interventions. Nevertheless, individuals with high self-efficacy characterized by strong direct communication may have skills for managing physical space. Indigenous people given voice in these studies demonstrated they can overcome known barriers, manage relationships, and successfully establish and maintain smoke-free homes [23,25].

To our knowledge, there has been only one peer-reviewed study evaluating a smoke-free home intervention for Indigenous populations. The study, included in this review, was a randomized controlled trial of behavioral ‘coaching’ about SHS, in addition to usual care, delivered during home visits following the birth of a new baby. The study was implemented in homes with at least one smoker in Darwin, Australia and Auckland, New Zealand. The control group received usual post-natal care. This intervention did not show a significant effect for the primary study outcome measure, which was the rate of presentations to a health provider for acute respiratory illness in the baby’s first year of life. Notably 94% of participants in the intervention group and 95% in regular post-care group identified their homes having full smoking bans at baseline, and at 12 months post intervention [36]. A qualitative study nested within this trial, also included in this review, found that women believed they were effective in protecting their children from SHS [35].

A systematic review of qualitative studies investigating smoke-free homes has been conducted [39]. Of the 22 included studies, only two included Indigenous people [23,25]. Seven analytical themes were developed including knowledge, skills, community norms, relationships, perceived benefits, addiction and practicalities. The review similarly reported that protecting children was a motivator for implementing smoke-free home rules and that concerns about maintaining relationships made it difficult to restrict smoking in the home. Our work uniquely focused on Indigenous populations, and together the two reviews provide a comprehensive review of barriers, motivators and enablers of smoke-free homes across Indigenous and non-Indigenous population groups. 

A strength of this review is that to our knowledge, it is the first systematic review to explore the implementation of smoke-free homes in Indigenous populations experiencing colonization and living within high-income countries. The included studies have been analyzed using a SCT lens to understand the influences and challenges of establishing smoke-free homes from a health promotion perspective. SCT was selected to demonstrate the utility of using this behavioral theory to potentially inform the design and development of smoke-free home research interventions. A limitation of the review is that the initial abstract screening was completed by a sole reviewer. Care was taken to ensure all studies were identified, however, there remains a risk that some papers may have been missed. The diversity of methodological approaches used in the studies was challenging. There are no agreed tools to systematically assess quality in mixed reviews, instead the articles have been summarized in Table 1. Data collection methods used in some studies may have had a differential effect on the reporting of smoke-free homes, resulting in reporting bias and unequal comparisons. One study that used telephone surveys for data collection consistently showed positive self-regulated responses, indicating that the participants limited smoking inside their homes [33]. However, other national data and studies using face-to-face interviews suggests that smoking inside the home is reported more frequently [2,3,35]. It is also worth noting that no studies focused specifically on the views of Indigenous men, and doing so may be useful for developing strategies targeted to Indigenous men in the home. This review has not captured successful smoke-free home projects that were reported in non-peer-reviewed literature. For example, in the Blue Light Project community members in Canadian Aboriginal reserves placed “blue-colored” lights (light bulbs) at the entrance of their homes to show visitors that their home was smoke-free. This project visited over 800 homes in a Canadian Aboriginal reserve and gave out over 300 “blue-colored” lights to self-declared smoke-free households [23]. These types of initiatives are important for increasing our understanding of successful implementation of community-based strategies.

Supporting the establishment of smoke-free homes has been shown to be effective for preventing the uptake and cessation of tobacco smoking in the general population, and may be a particularly useful intervention for Indigenous populations [15]. It was evident from this review that families, including those who live in disadvantaged circumstances, successfully implement smoke-free homes, and it is important to understand these characteristics, and promote them. Table 2 details our suggestions for policy, practice and research based on the range of personal, behavioral and environmental factors that influence the implementation of smoke-free homes. Due to the reciprocal nature of the factors, health promotion programs should consider a range of factors in the development of programs [40]. In terms of how to achieve or develop an intervention based on the above factors, using participatory processes by actively involving Indigenous people in developing culturally appropriate health promotion programs is vital [41].

## 5. Conclusions

This review of fifteen studies through a SCT lens identified influences and barriers to the establishment of smoke-free homes in Indigenous communities. It is important to support individuals and families to create smoke-free homes which may represent a catalyst to reducing long-term smoking behaviors by supporting cessation among established smokers and preventing uptake among adolescents [13,19]. The review elicited practical recommendations from the participants in the included studies, and the importance of relationships and role models, and how best to communicate home smoking rules [22,23,24,25,27,29,31,33]. Cultural considerations are paramount, and sharing was a common theme related to socializing and kinship [23,27,30,34]. In developing a suitable intervention, these considerations can be born in mind. The interplay of reciprocal determinism can be useful to inform the design and development of appropriate interventions. For example, by using a multilevel intervention that simultaneously addresses personal (building self-efficacy and collective efficacy through education and support for behavior change), behavioral (establishing smoke-free home behaviors and quitting) and supportive environmental (social and physical) influences that support smoke-free spaces. However, local factors and diversity of Indigenous population needs to be always taken into consideration.

## Figures and Tables

**Table 1:** Summary of characteristics of 15 included studies.

Study (First Author, Year) [Ref]	Location of Study by Country and Region as Described by Authors	Method	Sample	Total Participants *N*	Indigenous Participants *N*	Focus of Study
Arjunan et al., 2016 [32]	Australia Sydney	Quantitative—survey of Aboriginal community members	Community members	663	663	Associated factors with smoking, cessation behaviors and attitudes towards smoke-free homes. Indigenous specific focus
Bottorff et al., 2010 [23]	Canada Reserve	Qualitative—focus groups	Pregnant women, key informants, elders, youth	63	63	Explore what influences smoking bans in the home. Indigenous specific focus
Gillespie et al., 2005 [33]	New Zealand National	Quantitative—population telephone survey	Women, men	2731	924	Assess passive smoking exposure in homes and cars, and describe attitudes and behaviors towards smoke-free settings. Mixed ethnic population
Glover et al., 2006 [22]	New Zealand Auckland	Qualitative—focus groups and in-depth interviews	Parents and caregivers	61	6	Investigate parental attitudes and behaviors regarding their children’s uptake of smoking. Indigenous specific focus
Glover et al., 2013 [24]	New Zealand Auckland, Wellington	Qualitative—focus groups and in-depth interviews	Children	41	27	Describe attitudes of children with respiratory illness towards passive smoking, smoking and parental cessation. Mixed population
Glover et al., 2015 [31]	Australia & New Zealand Darwin & Auckland	Qualitative—semi-structured interviews nested within a randomized controlled trial	Mothers	26	26	Understand smoke-free rules around infants. Indigenous specific focus—Maori and Aboriginal
Gould et al., 2013 [25]	Australia Regional New South Wales	Qualitative—focus groups	Pregnant women, partners/family	18	18	Describe women’s and family-members’ attitudes and experiences of prenatal tobacco smoking and household smoking. Indigenous specific focus
Johnston & Thomas 2008 [34]	Australia Remote Northern Territory	Qualitative—semi-structured interviews	Community members, health staff	38	29	Understand motivators of smoking uptake, routine smoking behavior and motivators and issues related to quitting. Indigenous specific focus
Johnston et al., 2011 [26]	Australia Darwin & Remote Northern Territory	Quantitative—cross-sectional survey	Post-partum women	215	215	Describe trends in maternal smoking and smoking in the home. Indigenous specific focus
Kegler et al., 2002 [35]	United States Rural	Quantitative—in-home survey	Parents/ caregivers	380	167	Understand household and car smoking restrictions in low-income, rural Native American and White households with young children. Native American and white parents or guardians participated.
Robertson et al., 2013 [27]	Australia Remote Northern Territory	Multi-methods—community surveys, focus groups and in-depth interviews	Key informants, community members, health staff	400	400	Describe a grass-roots response to passive smoking in the community setting. Indigenous specific focus
Stevenson et al., 2013 [28]	Australia Remote Northern Territory	Quantitative—community survey	Community members	258	258	Comparison of those who restrict smoking in the home, car and workplace, and those who do not. Indigenous specific focus
Varcoe et al., 2010 [29]	Canada Rural reserve	Qualitative—individual, group interviews	Key informants	66	66	Describe influences on smoking practices and SHS exposure with a focus on pregnancy and children. Indigenous specific
Walker et al., 2015 [36]	Australia & New Zealand Darwin/Greater Darwin & Manukau region	Quantitative—data collection in participant homes	Mother/infant dyads	228	228	Evaluate a smoke-free intervention with acute respiratory related visits to a health care provider in the infant’s first year of life as the main outcome measure. Indigenous specific focus—Both Maori and Aboriginal
Wood et al., 2008 [30]	Australia Perth	Qualitative—focus groups and in-depth interviews	Pregnant women & health workers	50	50	Investigate the cultural context of tobacco smoking relating to smoking in pregnancy. Indigenous specific focus

**Table 2:** Recommended policy, practice and research strategies.

SCT	Themes	Recommendations for Policy and Practice
Personal factors	Knowledge of the health impact of SHS and strong beliefs about potential harm to exposed to children Self and collective efficacy to implement smoke-free homes	Strategies to support families transfer knowledge of the importance of SFH into practice by supporting individuals and families develop a discourse to help with the decision-making process for implementing smoke-free homes Health promotion messages co-developed with Indigenous communities can be positively framed as family and community interventions.
Behavioral factors	Smoking behaviors in and round the home Success stories	Identifying change agents and leaders in the community to role model and promote smoke-free home behaviors Narrative and video-based media to promote success stories
Environmental factors	Social and physical management of the home	Understanding decision-making processes, and the power structure of relationships within family homes may improve the development of smoke-free home intervention strategies Future qualitative and quantitative research could explore the how to build self and collective efficacy in the decision-making processes for implementing smoke-free homes in Indigenous community setting, rather than focusing only on the individual smoker

SCT: Social Cognitive Theory.
